# ECMO-TO-GO: Application of a portable on the body veno-arterial ECMO device in a bridge-to-transplantation setting

**DOI:** 10.1016/j.jhlto.2024.100069

**Published:** 2024-02-17

**Authors:** Bastian Schmack, Jasmin S. Hanke, Jan D. Schmitto, Christian Kühn, Arjang Ruhparwar

**Affiliations:** Department of Cardiac-, Thoracic-, Transplantation and Vascular Surgery, Hannover Medical School, Hannover, Germany

**Keywords:** temporary mechanical assist device, extracorporeal membrane oxygenation, ECMO, mobilization, TandemHeart, Voyager Vest, bridge to transplantation

## Abstract

Extracorporeal membrane oxygenation (ECMO) is a well-established therapy to bridge heart- and lung-failure patients to recovery, transplantation, or permanent assist devices. However, it is often associated with thorough patient immobility resulting in an increased hospital length-of-stay and inferior outcomes. To address the challenge of immobility on temporary mechanical support system (tMCS), the “ECMO TO GO” concept was developed to offer full patient mobilization. We present the successful application of a wearable ECMO device. With this setup, the patient was successfully and recurrently mobilized and could walk >200 m on hospital floors. No complications or adverse events occurred during the mobilization procedure.

Extracorporeal membrane oxygenation (ECMO) is a well-established therapy to bridge heart- and lung-failure patients to recovery, transplantation, or permanent assist devices. However, it is associated with immobility and subsequent loss of muscle mass, critical illness polyneuropathy, impaired gastrointestinal function as well as risk of pneumonia and psychological disorders, resulting in an increased hospital length-of-stay and inferior outcomes.^1^, ^2^, ^3^, ^4^, ^5^ To address the challenge of immobility on temporary mechanical support system (tMCS), the “ECMO-TO-GO” concept was developed to offer hands-free full patient mobilization. We present the application of a wearable ECMO device.

A 33-year-old female, who suffered from severe hypertrophic cardiomyopathy resulting in end-stage biventricular failure, was listed for cardiac transplantation. The patient was initially supported with percutaneous veno-arterial extracorporeal membrane oxygenation (va-ECMO) support via femoral access due to acute deterioration of cardiac function. Due to biventricular failure, we refrained from the implantation of a permanent assist device and decided on a bridge-to-transplantation strategy. After 6 days on tMCS support, we implemented an upper-body-cannulation concept via 17-Fr-Bio-Medicus cannula (Medtronic) in the right superior jugular vein and a 15-Fr-HLS cannula (Maquet, Germany) via 6 mm Dacron Uni-Graft (B. Braun, Germany) to the axillary artery to avoid immobilization of the patient. Distal arm perfusion was performed through a 6-Fr-cannula. A TandemHeart driver-unit (LivaNova, UK) and TandemLung oxygenator (LivaNova, UK) were used. For mobilization, the patient wore a Voyager Vest (TM oder (LivaNova, UK), which facilitates convenient carrying of the ECMO equipment (Figures 1 and 2). For anticoagulation, the activated partial thromboplastin time (aPTT) target was 50 to 60 seconds. With this setup, the patient was successfully and recurrently mobilized and could walk >200 m on hospital floors (Online supporting material, Video 1). No complications or adverse events occurred during the mobilization procedure. The ECMO oxygenator was exchanged due to decreasing function caused by thrombosis after 20 and 21 days on device. Ultimately, the patient was successfully transplanted after 30 days on ECMO support.

**Figure 1:**
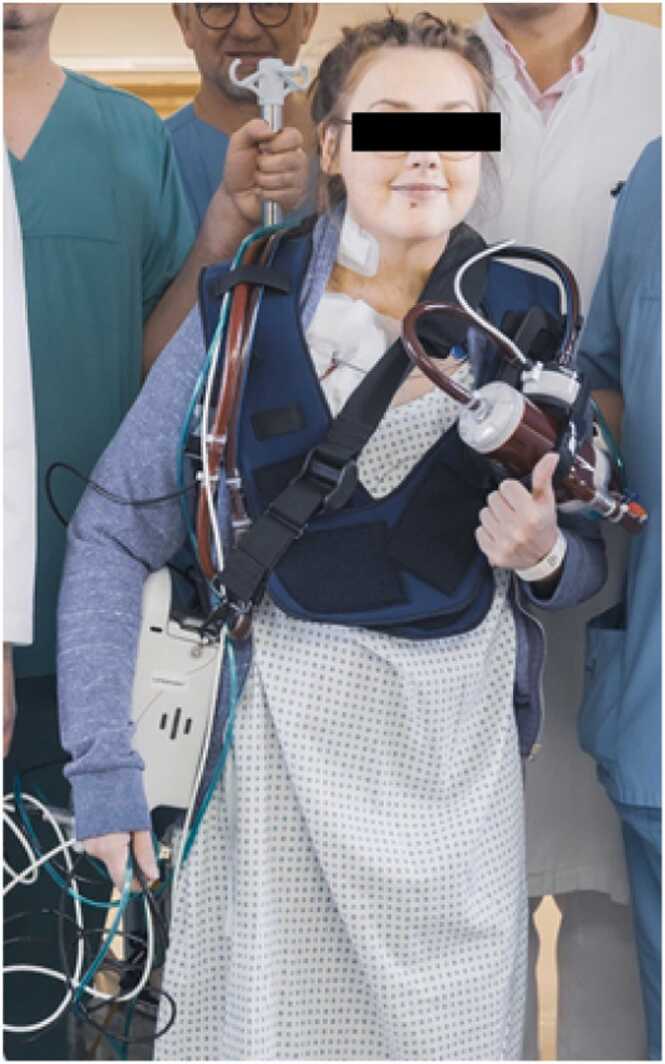
Mobilization with the “ECMO-TO-GO” concept. The patient wears the Voyager Vest (LivaNova PLC, UK), which offers the possibility to carry the ECMO power unit as well as the oxygenator. ECMO, extracorporeal membrane oxygenation.

**Figure 2:**
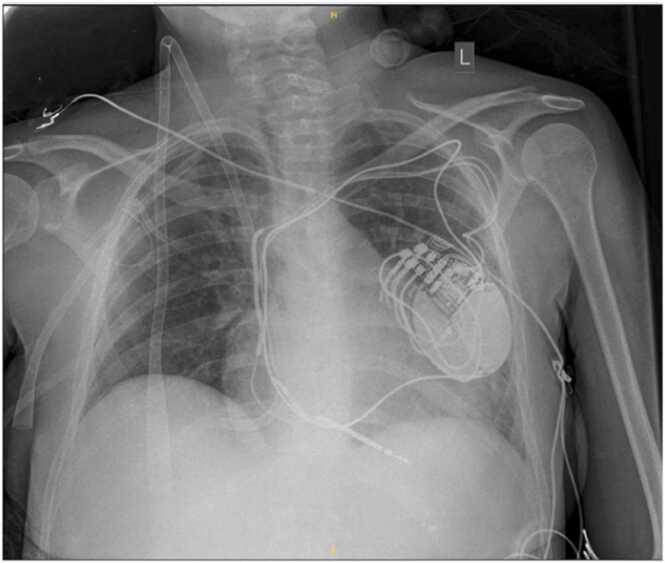
X-ray of patient supported by the “ECMO-TO-GO” concept.

Supplementary material related to this article can be found online at doi:10.1016/j.jhlto.2024.100069.

The following is the Supplementary material related to this article Video S1.

Video S1 Video 1 Patient on ECMO support..

The wearable support strategy “ECMO-TO-GO” allows for excellent mobilization and enables patients to increase physical activity, possibly avoiding muscular atrophy and decreasing risks associated with long-term immobilizations, such as infection and mental disorders.^1^, ^2^, ^3^, ^4^, ^5^ The importance of physical activity of ECMO patients for preconditioning has been described in various publications.^1^, ^2^, ^3^, ^4^, ^5^ In the setting of percutaneous tMCS, the use of bed bikes or other standing-hospital-beds avoids strain on the femoral access site.^1^, ^2^, ^3^ Nevertheless, these strategies are limited and do not allow full mobilization. Contrarily, upper-body-cannulation offers the advantage of avoidance of vessel access in the groin and hence permits full leg movement. However, disadvantageous is the need for surgery for the application of upper-body-cannulation. Compared to conventional upper-body-cannulation, the novelty of the “ECMO-TO-GO” approach is the smaller equipment, which can be carried with the Voyager Vest. Thus, there is no need for further external equipment possibly serving as tripping hazards and offering patients increased freedom of movement. The oxygenator that was used in the presented case (TandemLung, LivaNova, UK) will be discontinued in 2024. Nevertheless, we promote a modular system, which can be used in various technical ECMO setups.

The “ECMO-TO-GO” concept is a feasible and effective tool for full mobilization on ECMO support. It is recommended for active patients, who are expected to have increased waiting times for cardiac transplantation and benefit from comprehensive continuous physical therapy. Increased use of the “ECMO-TO-GO” concept might lead to improved surgical preconditioning owing to avoidance of muscular atrophy and reduction of risk factors associated with long-term immobilization.

## Disclosure statement

The authors declare that they have no known competing financial interests or personal relationships that could have appeared to influence the work reported in this paper.

No funding was received for this study.

## Patient consent

The patient gave written consent for the publication of the video material.
